# The repeatability and consistency of different methods for measuring the volume parameters of the primary rectal cancer on diffusion weighted images

**DOI:** 10.3389/fonc.2023.993888

**Published:** 2023-03-09

**Authors:** Yong-juan Qiu, Lin-Li Zhou, Jun Li, Yi-fei Zhang, Yong Wang, Yan-song Yang

**Affiliations:** Department of Radiology, Affiliated Tumor Hospital of Nantong University, Nantong, Jiangsu, China

**Keywords:** rectal cancer, repeatability, consistency, volume, semi-automatic delineation

## Abstract

**Background:**

To determine the reproducibility of measuring the gross total volume (GTV) of primary rectal tumor with manual and semi-automatic delineation on the diffusion-weighted image (DWI), examine the consistency of using the same delineation method on DWI images with different high b-values, and find the optimal delineation method to measure the GTV of rectal cancer.

**Methods:**

41 patients who completed rectal MR examinations in our hospital from January 2020 to June 2020 were prospectively enrolled in this study. The post-operative pathology confirmed the lesions were rectal adenocarcinoma. The patients included 28 males and 13 females, with an average age of (63.3 ± 10.6) years old. Two radiologists used LIFEx software to manually delineate the lesion layer by layer on the DWI images (b=1000 s/mm^2^ and 1500 s/mm^2^) and used 10% to 90% of the highest signal intensity as thresholds to semi-automatically delineate the lesion and measure the GTV. After one month, Radiologist 1 performed the same delineation work again to obtain the corresponding GTV.

**Results:**

The inter- and intra-observer interclass correlation coefficients (ICC) of measuring GTV using semi-automatic delineation with 30% to 90% as thresholds were all >0.900. There was a positive correlation between manual delineation and semi-automatic delineation with 10% to 50% thresholds (P < 0.05). However, the manual delineation was not correlated with the semi-automatic delineation with 60%, 70%, 80%, and 90% thresholds. On the DWI images with b=1000 s/mm^2^ and 1500 s/mm^2^, the 95% limit of agreement (LOA%) of measuring GTV using semi-automatic delineation with 10%, 20%, 30%, 40%, 50%, 60%, 70%, 80%, and 90% thresholds were -41.2~67.4, -17.8~51.5, -16.1~49.3, -26.2~50.1, -42.3~57.6, -57.1~65.4, -67.3~66.5, -101.6~91.1, -129.4~136.0, and -15.3~33.0, respectively. The time required for GTV measurement by semi-automatic delineation was significantly shorter than that of manual delineation (12.9 ± 3.6s vs 40.2 ± 13.1s).

**Conclusions:**

The semi-automatic delineation of rectal cancer GTV with 30% threshold had high repeatability and consistency, and it was positively correlated with the GTV measured by manual delineation. Therefore, the semi-automatic delineation with 30% threshold could be a simple and feasible method for measuring rectal cancer GTV.

## 1 Introduction

Rectal cancer is a malignant neoplasm of the gastrointestinal tract with high incidence and mortality rates. The Global Cancer Statistics 2020 report estimates that there are approximately 732,210 new cases of rectal cancer and 339,022 deaths worldwide in 2020 (1). Tumor volume can quantitatively reflect tumor burden, and it is related to tumor stage and survival rate (2). Moreover, the therapeutic effect can be quantitatively evaluated based on the change of tumor volume before and after treatment (3–5). Therefore, it is critical to measure the tumor volume accurately and conveniently. Tumor volume can be measured by CT, MR, PET/CT, and other imaging examinations. In previous studies on tumor volume measurement with CT and MR, the tumor lesions were manually delineated layer by layer to obtain the tumor volume of each level (2D area multiplied by the layer thickness), and the gross tumor volume (GTV) was obtained by adding all the 2D volumes of the entire lesion (6–9). The manual outlining method of measuring GTV does not accurately differentiate tumor tissue from inflammatory response, post-treatment fibrosis and residual tumor. In addition, it is time consuming to outline tumor lesions layer by layer manually and it has poor intra- and inter-observer agreement (10).

Recently, many studies suggest that the volume parameters of the primary rectal cancer on MR images have great potential in predicting lymph node metastasis, vascular invasion, and evaluating the efficacy of radiotherapy and chemotherapy (11–15). And all these studies showed that the volume parameter on DWI images were better than the volume parameters on T2WI. Using PET images, the metabolic tumor volume (MTV) can be semi-automatically delineated and measured based on the percentage threshold of the maximum standardized uptake value (SUVmax) or the maximum standardized uptake value normalized to lean body mass (SULmax) of the primary tumor. This semi-automatic delineation method has high feasibility and reproducibility, and is widely used in clinical work and scientific research (16, 17). The lesion and background on the PET images and DWI images have high contrast. Based on the semi-automatic delineation method of the tumor lesion on PET image, this study explored the feasibility of semi-automatic delineation of primary rectal cancer lesion on DWI images. The thresholds were selected based on different percentages of the maximum signal intensity of the lesion. We also discussed the reproducibility of GTV measurement by manual and semi-automatic delineation of the primary rectal cancer on DWI, as well as the consistency of using the same delineation method to obtain GTV on the DWI images with different high b-values. Based on the findings, we proposed the best GTV delineation method for rectal cancer, which can be applied in clinics and related research.

## 2 Materials and methods

### 2.1 Study subjects

This study is a prospective study, approved by the Ethics Committee of Nantong Tumor Hospital. The approval number was 2019-075.

**Figure 1:**
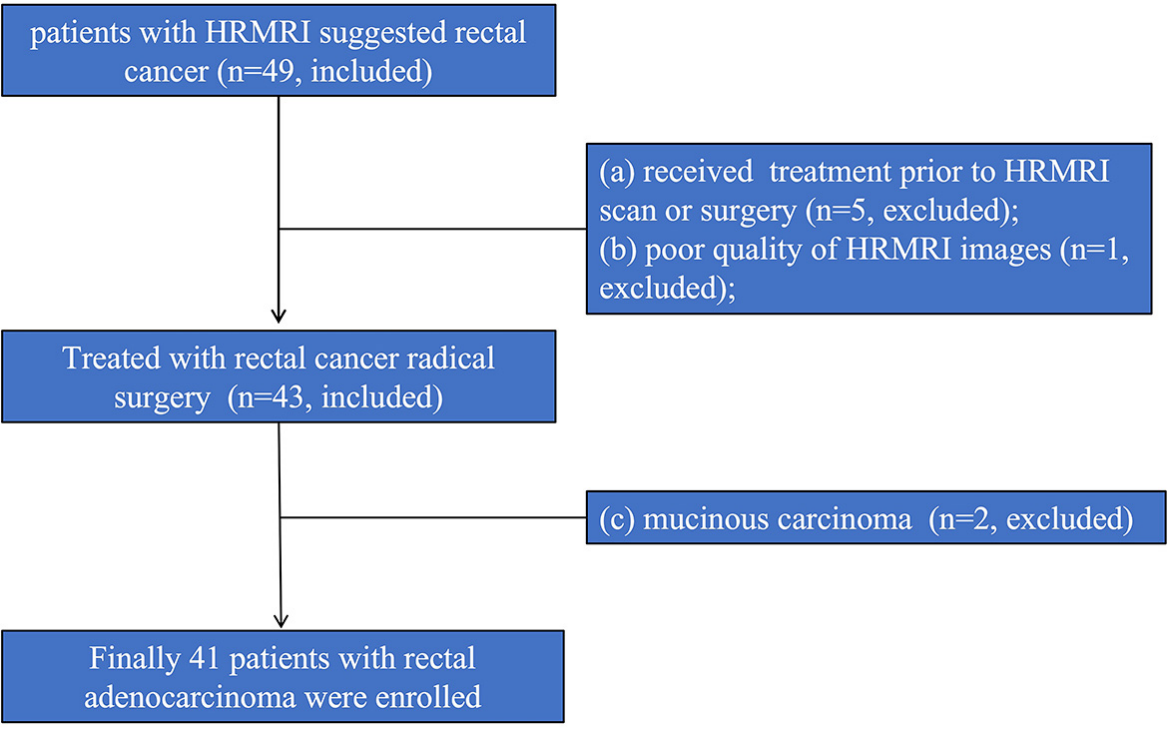
Flow chart of the study population.

The patients diagnosed with rectal cancer by high-resolution magnetic resonance imaging (HRMRI) in our hospital from January 2020 to June 2020 were prospectively enrolled. Inclusion criteria: (1) HRMRI was performed within one week before surgery; (2) rectal cancer was confirmed by pathology after surgery. Exclusion criteria: (1) Since more than 50% of the mucinous adenocarcinoma lesions consist of extracellular mucus, the tumor cell density is low, the diffusion limitation is not obvious, the apparent diffusion coefficient (ADC) value is high, and it is difficult to delineate the lesion; therefore, the mucinous adenocarcinoma cases diagnosed by postoperative pathology were excluded (18); (2) Radiotherapy and chemotherapy were performed before surgery; (3) The image quality was poor and it was difficult to delineate the lesion. Finally, 41 patients were enrolled in this study, and 8 were excluded. The excluded cases included: 2 cases with mucinous adenocarcinoma; 5 cases with preoperative radiotherapy and chemotherapy; and 1 case with poor image quality. The enrollment procedure of the study population is shown in **Figure 1**.

### 2.2 MRI examination and methods

Before the examination, patients were fasted for more than 4 hours, and their intestines were cleaned with Glycerol Enema. The Siemens MAGNETOM Verio 3.0 superconducting MR scanning machine and 8-channel body surface phased array coil were used for MRI examination. The patient was placed in a supine position with the head entered first. The center of the coil was placed on the same level with symphysis pubis, and the necessary adjustments were made according to tumor location. The MRI sequence used a two-dimensional fast spin echo sequence without fat suppression, and its parameters were as follows: layer thickness 3 mm; layer interval 0.3 mm, TR/TE: 2500~3500ms/100ms; field of view: 18cm×18cm; pixel matrix: 320× 320; ETL: 29. The scanning sequence was: sagittal position, oblique axial position perpendicular to the long axis of the lesion, oblique coronal position parallel to the anal canal, axial T1-weighted fast gradient echo sequence, axial diffusion-weighted imaging (DWI) sequence (b=50, 1000, 1500 s/mm^2^), and enhanced scan (including arterial phase and venous phase) (19).

### 2.3 Image delineation

**Figure 2:**
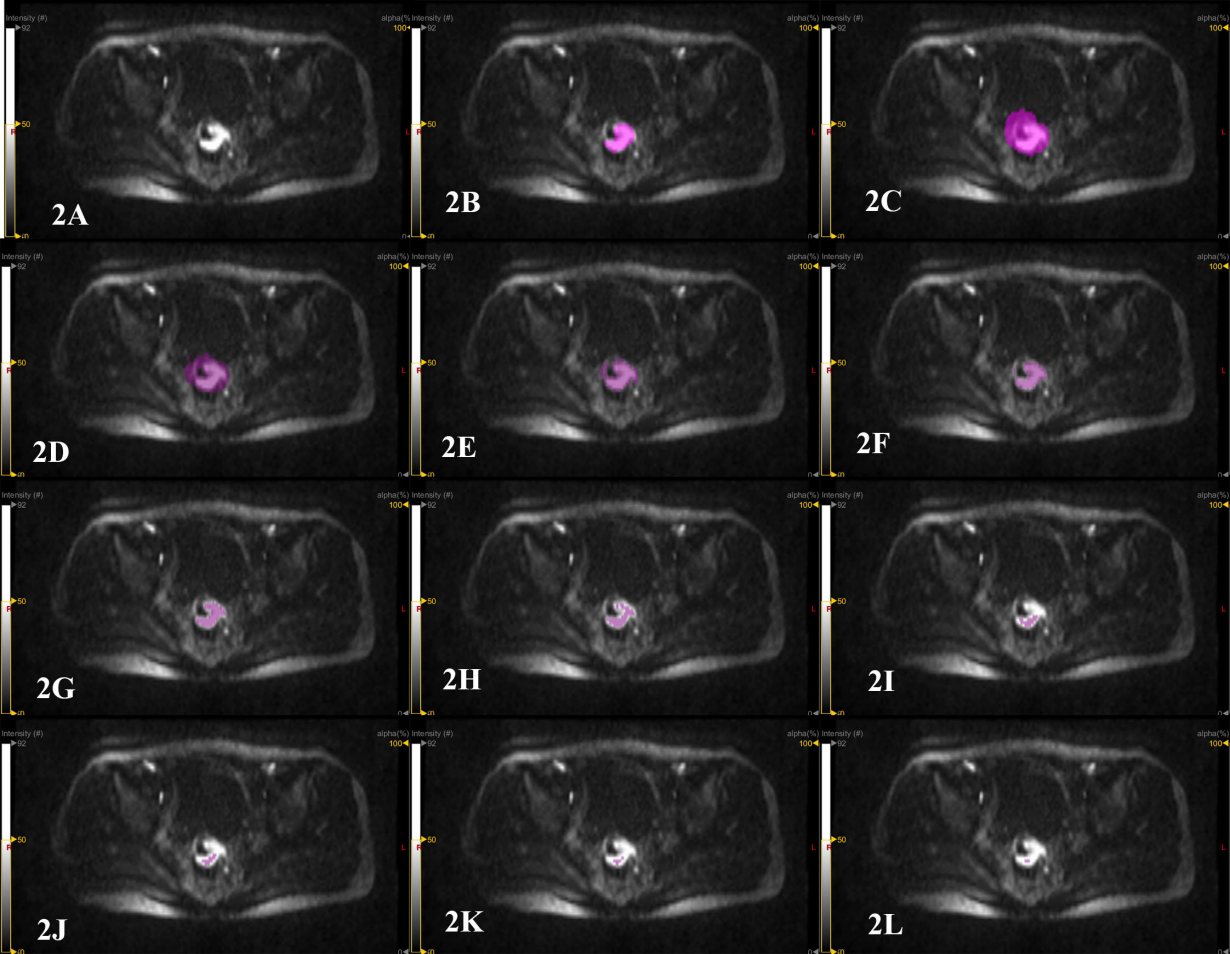
Patient, male, 67 years old, with distal rectal cancer. **(A)** the original image of the primary rectal cancer on a certain layer of the DWI image with b=1500 s/mm^2^. The high signal area is the tumor area. **(B)** manual delineation of the primary lesion of rectal cancer on this layer using the pencil 2D tool in the LIFEx software to measure GTV. The LIFEx software shows that the volume of the rectal tumor on this level is 4.078 cm^3^. After the lesion is delineated layer by layer, the LIFEx software automatically integrated the tumor volume of each layer, and the GTV was 22.25 cm^3^. **(C)** semi-automatic delineation of the primary lesion of rectal cancer by using the circle 3D tool of LIFEx software to cover the entire area of the primary rectal cancer. **(D–L)** semi-automatic delineation of tumor lesions using 10%, 20%, 30%, 40%, 50%, 60%, 70%, 80% and 90% of the highest signal intensity in the area as thresholds. The measured GTV were 70.29 cm^3^, 34.52 cm^3^, 19.16 cm^3^, 10.52 cm^3^, 5.564 cm^3^, 1.813 cm^3^, 0.705 cm^3^, 0.252 cm^3^ and 0.101 cm^3^.

The open-source software LIFEx (https://www.lifexsoft.org/) was used to complete the lesion delineation on the high b DWI (b=1000 s/mm2 and 1500 s/mm2). Manual delineation: Compared with the low and medium signal areas adjacent to the rectal wall or pelvic background, the high signal area was determined as the primary lesion of rectal cancer (**Figure 2A**). The primary lesion of rectal cancer was manually delineated layer by layer using the pencil 2D tool in LIFEx software (**Figure 2B**) to obtain the tumor volume per layer (2D area multiplied by layer thickness) and the LIFEx software automatically integrated all 2D volumes of the entire lesion to obtain the GTV. The high signal structures on DWI image such as bladder, surrounding large lymph nodes, blood vessels, and seminal vesicles were avoided during the delineation process. Semi-automatic delineation: the circle 3D tool in the LIFEx software was used to cover the primary lesion of rectal cancer. The high signal structures on the DWI image, such as bladder, surrounding large lymph nodes, blood vessels, and seminal vesicles were avoided (**Figure 2C**). The lesions were semi-automatically delineated using 10%, 20%, 30%, 40%, 50%, 60%, 70%, 80%, and 90% of the highest signal intensity in the area as the thresholds, and the corresponding GTV were obtained (**Figures 2D–L**). Two radiologists (with 16 years and 5 years of experience in abdominal MR radiology) completed the delineation work within two weeks. One month later, radiologist 1 performed the delineation work again and measured the GTV (20).

### 2.4 Statistical analysis

SPSS25.0 software was used to analyze the data. The GTV measured by various methods was a continuous variable. Interclass correlation coefficients (ICC) were used to evaluate the repeatability of various GTV measurements, and ICC>0.9 was used as the standard to identify methods with good repeatability. The Bland-Altman chart method was used to analyze the consistency of GTV measurement on DWI images with b= 1500 s/mm^2^ and b= 1000 s/mm^2^. The distribution of GTV measured by various methods was tested by Shapiro-Wilk test to determine whether it was normally distributed, and the correlation of GTV measured on the DWI image with b=1000 and 1500 s/mm^2^ was analyzed by Spearman correlation analysis. T-test was used to compare the time required for manual delineation and semi-automatic delineation. P<0.05 was considered statistically significant.

## 3 Results

### 3.1 General information and postoperative pathological results

Among the 41 enrolled patients with rectal adenocarcinoma, 28 were males and 13 were females; the average age was 63.3 ± 10.6 years old (33-83 years old). There were 21 cases with upper rectal tumors, 9 cases with middle rectal tumors, and 11 cases with lower rectal tumors. Postoperative pathology showed that 8 cases were poorly differentiated tumors, 24 cases were moderately differentiated tumor, and 9 cases were highly differentiated; there were 4 cases in T1 stage, 8 cases in T2 stage, and 28 cases in T3 stage; moreover, 21 cases were in N0 stage, 12 cases were in N1 stage, and 8 cases were in N2 stage.

### 3.2 The repeatability of manual delineation and semi-automatic delineation for measuring GTV

On the DWI image with b value of 1500 s/mm^2^ or 1000 s/mm^2^, the intra- and inter-observer ICC for measuring GTV using semi-automatic delineation with 30%, 40%, 50%, 60%, 70%, 80%, and 90% of the highest signal intensity in the primary rectal cancer area as thresholds were all > 0.900. At b= 1500 s/mm^2^, the intra- and inter-observer ICC of semi-automatic delineation with 10% and 20% as thresholds were 0.496, 0.785, 0.906, and 0.936, respectively; at b= 1000 s/mm^2^, the corresponding ICC were 0.624, 0.570, 0.894, and 0.795, respectively. The intra- and inter-observer ICC of manual delineation for measuring GTV were 0.736, 0.632, 0.883, and 0.605 (**Table 1**).

**Table 1:** Repeatability of semi-automatic delineation and manual delineation for measuring GTV (n=41).

	b= 1500 s/mm^2^				b= 1000 s/mm^2^			
Measurement methods	Intra-observer ICC	95% CI	Inter-observer ICC	95% CI	Intra-observer ICC	95% CI	Inter-observer ICC	95% CI
10%	0.496	0.091~0.811	0.785	0.633~0.879	0.624	0.072~0.865	0.570	0.313~0.747
20%	0.906	0.582~0.966	0.936	0.881~0.966	0.894	0.682~0.955	0.795	0.597~0.894
30%	0.985	0.954~0.993	0.966	0.931~0.983	0.962	0.921~0.981	0.922	0.829~0.961
40%	0.995	0.990~0.997	0.985	0.970~0.992	0.983	0.968~0.991	0.968	0.933~0.984
50%	0.999	0.997~0.999	0.997	0.995~0.999	0.994	0.989~0.997	0.993	0.985~0.996
60%	1.000	1.000~1.000	1.000	1.000~1.000	0.999	0.998~0.999	0.999	0.998~0.999
70%	1.000	0.999~1.000	1.000	1.000~1.000	1.000	1.000~1.000	1.000	1.000~1.000
80%	1.000	0.999~1.000	1.000	1.000~1.000	1.000	1.000~1.000	0.993	0.988~0.996
90%	0.999	0.998~0.999	0.999	0.998~0.999	0.998	0.997~0.999	0.998	0.997~0.999
Manual delineation	0.736	0545~0.853	0.632	0.028~0.850	0.883	0.787~0.937	0.605	0.153~0.812

### 3.3 The correlation between manual delineation and semi-automatic delineation for measuring GTV

By using the average value of the GTV measurements from the two manual measurements by Radiologist 1 and the one manual measurement by Radiologist 2 as the reference, we analyzed the correlation between manual delineation and semi-automatic delineation with different thresholds The Shapiro-Wilk test showed that the data measured using different methods were all non-normally distributed. Spearman analysis showed that on DWI image with b=1500 s/mm^2^, there was a positive correlation between manual delineation and semi-automatic delineation with 10%, 20%, 30%, 40%, and 50% thresholds (Spearman’s correlation coefficients were 0.839, 0.847, 0.820, 0.711, 0.529, and all P < 0.05), and there was no correlation between manual delineation and semi-automatic delineation with 60%, 70%, 80%, 90% thresholds (Spearman’s correlation coefficients were 0.295, 0.134, 0.043, 0.162, and all P > 0.05); on the DWI image with b= 1000 s/mm^2^, there was a positive correlation between manual delineation and semi-automatic delineation with 10%, 20%, 30%, 40%, and 50% thresholds (Spearman’s correlation coefficients were 0.827, 0.774, 0.705, 0.630, 0.483, all P < 0.05), and there was no correlation between manual delineation and semi-automatic delineation with 60%, 70%, 80%, 90% thresholds (Spearman’s correlation coefficients were 0.163, 0.021, -0.073, -0.094, all P > 0.05). This result indicated there was a positive correlation between semi-automatic measurement of GTV and manual outlining at thresholds of 10%, 20%, 30%, 40% and 50%.

### 3.4 The consistency between semi-automatic delineation and manual delineation for measuring GTV on DWI images with different b values

**Figure 3:**
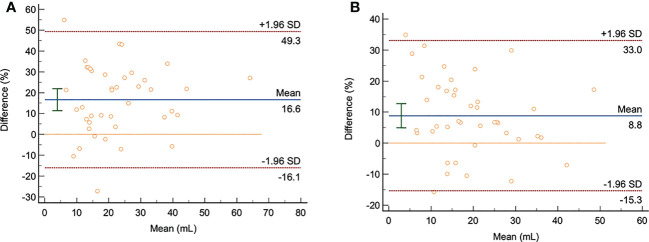
The Bland-Altman analysis chart of the semi-automatic and manual delineation for measuring GTV on the DWI images with b= 1000 s/mm^2^ and b= 1500 s/mm^2^ (n=41). **(A, B)**: The consistency analysis of GTV measurement by radiologist 1 using manual delineation and semi-automatic delineation with 30% as thresholds on the DWI images with b= 1000 s/mm2 and b= 1500 s/mm2. The averaged difference was 16.6 and 8.8; the 95% LOA were -16.1~49.3 and 15.3~33, respectively.

Bland-Altman (**Figure 3**) showed that: on the DWI images with b= 1000 s/mm^2^ and b= 1500 s/mm^2^, the average differences between manual delineation and semi-automatic delineation with 10%, 20%, 30%, 40%, 50%, 60%, 70%, 80%, and 90% of the highest signal intensity in rectal cancer as thresholds were 13.1, 16.8, 16.6, 11.9, 7.6, 4.2, -0.4, -5.2, 3.3, and 8.8, respectively; the 95% limits of agreement (LOA) were -41.2~67.4, -17.8~51.5, -16.1~49.3, -26.2~50.1, -42.3~57.6, -57.1~65.4, -67.3~66.5, -101.6~91.1, -129.4~136.0, -15.3~33.0, respectively.

Of the upper and lower LOA ranges with thresholds of 10% to 50%, the upper and lower LOA ranges with a threshold of 30% are the smallest and within the clinically acceptable range with good consistency. On the other hand, the semi-automatic delineation with 50%, 60%, 70%, 80% and 90% thresholds had a large 95% LOA, which was not acceptable in clinic, and the consistency was poor.

### 3.5 The time comparison between manual and semi-automatic delineation for measuring GTV

The time required for semi-automatic delineation was significantly lower than that of manual delineation to measure GTV (12.9 ± 3.6 vs 40.2 ± 13.1s). Therefore, compared with manual delineation, semi-automatic delineation requires less time to measure GTV, representing a simple and feasible method.

## 4 Discussion

The tumor volume parameter GTV of the primary rectal cancer can predict lymph node metastasis, vascular invasion, and evaluate the efficacy of radiotherapy and chemotherapy. Previous studies all used manual delineation method to outline the lesion layer by layer (11–15); however, this method has limitations for measuring GTV (10): (1) Inflammatory peritumoral response may affect the precise distinction between tumor and surrounding tissue; (2) It is difficult to distinguish between treatment-induced fibrosis and residual tumor; (3) Manual delineation is time-consuming and labor-intensive; moreover, the consistency and repeatability between observers are low due to the varying levels of experience of radiologists. Therefore, based on calculation method of MTV on PET, we proposed to use the percentage of the highest signal intensity of the tumor lesion area on the DWI image as the threshold to delineate the lesion semi-automatically. We calculated the repeatability and consistency of semi-automatic delineation with different thresholds and the correlation with manual delineation for measuring GTV, to propose the best delineation method for measuring GTV of rectal cancer. This study provided a reliable method for the measurement of GTV in future rectal cancer research.

Our results showed that the intra- and inter-observer ICCs of semi-automatic delineation with 30%, 40%, 50%, 60%, 70%, 80%, 90% thresholds were all >0.900 (10), and the intra- and inter-observer ICCs of manual delineation and semi-automatic delineation with 10%, 20% thresholds were <0.900. This might be due to that the semi-automatic delineation with 10% and 20% thresholds couldn’t eliminate the background signal in the pelvic cavity, and the manual delineation has inherent limitations (10).

Our results suggest that on the DWI images with b=1000 s/mm^2^ and 1500 s/mm^2^, the average differences between manual delineation and semi-automatic delineation with 30%, 40%, 50%, 60%, 70%, 80%, 90% thresholds, as well as 95% LOA and the proportion of points outside 95% LOA line were all relatively small and within the clinically acceptable range, while the 95% LOA between manual delineation and semi-automatic delineation with 10% and 20% thresholds were large, which were not acceptable in the clinic. This study also found that there was a positive correlation between manual delineation and semi-automatic delineation with 10%, 20%, 30%, 40%, and 50% thresholds, which can reflect the tumor size to a certain extent, while manual delineation was not correlated with semi-automatic delineation with 60%, 70%, 80%, 90% thresholds, suggesting that the semi-automatic delineation with 60%, 70%, 80%, 90% thresholds could not objectively reflect tumor size. Combining the results of repeatability and consistency, the semi-automatic delineation with 30%, 40%, 50% thresholds are good choices for measuring GTV. Moreover, after considering the correlation with manual delineation and to include as much tumor area as possible, we concluded that the semi-automatic delineation with 30% threshold was the best choice.

This conclusion is different from the 50% threshold for measuring MTV of advanced gastrointestinal malignancies on PET images proposed by Frings et al. (21) and the 40% threshold proposed by the European Association for Nuclear Medicine (17). This difference may be related to the different principles of the two imaging methods. PET is based on the metabolic rate of tumor lesions, while the principle of DWI is based on the increased density of tumor lesions and the reduced space between cells limiting the Brownian motion of water molecules.

In addition, this study also found that the time required for semi-automatic delineation of GTV measurement was significantly lower than that of manual delineation (12.9 ± 3.6 vs 40.2 ± 13.1s), and thereby it is an easier method.

Our study also has certain limitations: (1) This study is a single-center study with a relatively small sample size, and the measured GTV was non-normally distributed; thus, there was a certain selection bias; (2) Our study was done on a 3.0T Siemens MR machine. The repeatability and consistency between machines of different manufacturers and different field strengths (1.5T, 3.0T) have not been studied. (3) We found that when semi-automatic outlining was performed at the threshold of 30%, it showed significantly larger or smaller tumor volumes than manual outlining. This result may be due to the inflammatory response or necrosis around the tumor, which limits the ability of semi-automatic outlining. We will investigate this phenomenon further in a subsequent study.

In summary, using semi-automatic delineation with 30% of the highest signal intensity in primary rectal lesion area as the threshold to measure GTV has high repeatability and consistency, and there is a positive correlation with manual delineation. Therefore, it could be a simple and feasible method for measuring GTV of rectal cancer.

## 5 Conclusion

Based on its correlation with manual delineation, we found that the semi-automatic delineation with 30% threshold could be the best delineation method to measure GTV of primary rectal tumor on the diffusion-weighted image (DWI).

## Data availability statement

The original contributions presented in the study are included in the article/**Supplementary Material**. Further inquiries can be directed to the corresponding authors.

## Ethics statement

The studies involving human participants were reviewed and approved by the institutional ethics committee of Nantong Tumor Hospital. The patients/participants provided their written informed consent to participate in this study. Written informed consent was obtained from the individual(s) for the publication of any potentially identifiable images or data included in this article.

## Author contributions

Y-SY and Y-JQ contributed to conception and design of the study. L-LZ and JL organized the database. Y-SY and Y-JQ performed the statistical analysis. Y-JQ wrote the first draft of the manuscript. Y-FZ and YW wrote sections of the manuscript. All authors contributed to the article and approved the submitted version.
